# Tritium uptake in crops in the area with a high level of atmospheric tritium oxide in the territory of the former Semipalatinsk test site

**DOI:** 10.1371/journal.pone.0308959

**Published:** 2024-10-10

**Authors:** Yelena Polivkina, Yelena Syssoyeva, Axana Ivanova, Andrey Panitskiy, Laura Kenzhina, Valeriy Monaenko

**Affiliations:** Department of Radioecological and Biodosimetric Research, Brunch "Institute of Radiation Safety and Ecology" of National Nuclear Center of Republic of Kazakhstan, Kurchatov, Abay region, Kazakhstan; Universiti Teknologi Malaysia, MALAYSIA

## Abstract

During the period from 2019 to 2021, a series of experiments were carried out to study the uptake of tritium by crops in an area heavily contaminated with atmospheric tritium oxide (HTO), at the former Semipalatinsk test site in Kazakhstan. A quantitative assessment is given of the tritium uptake by typical crops (lettuce, tomatoes, peppers and beans) cultivated all over Kazakhstan in the case of a short-term tritium oxide vapor exposure. The plant samples were collected during and after exposure and analyzed for the tritium concentration in two chemical forms: tissue-free water tritium (TFWT) and organically bound tritium (OBT). During the entire series of experiments, the tritium concentration in free water from leaves and ambient air was of the same order of magnitude. The tissue water tritium concentrations of stems and edible parts was 1 to 2 orders of magnitude lower than in the surrounding air. The average value of the TFWT/HTO_atm_ ratio in the leaves and the edible part was (0.73±0.2) and (0.04±0.002), respectively. The organically-bound tritium concentration is 1–2 orders of magnitude lower than the tissue water tritium and ambient air concentrations. Under aerial tritium oxide uptake, the distribution of tritium in non-leafy crops was as follows: leaf–stem–fruit (in decreasing order). After exposure, a non-significant amount of tritium is firmly retained in plants for a long time. The tissue water tritium concentrations correlate closely with atmospheric tritium oxid (*r* = 0.76), correlate weakly with temperature (*r* = 0.43) and relative humidity (*r* = -0.43), and correlate moderately with solar radiation intensity (*r* = 0.56). There was no reliable correlation between the concentration of tritium in organic matter and in ambient air. The concentration of tritium in the free water of leaves is closely correlated with the concentration in the free water of the stems (*r* = 0.95) and fruits (*r* = 0.78). The organically-bound tritium concentration in leaves is closely correlated with the organically-bound tritium concentration in stems (*r* = 0.99) and fruits (*r* = 98). The results of the study should be considered when evaluating the impact of tritium oxide emissions on the population living near nuclear power.

## Introduction

Uptake of tritium (^3^H) by edible plants is an important pathway in transferring tritium to the human body [1,2]. Currently, the main source of tritium in the environment is routine release from nuclear fuel cycle facilities (NFC), in particular nuclear power plants [3,4]. As is known, the majority of tritium in the environment exists as tritiated water (HTO) [4–7]. This form of tritium has exceptional migration activity and bioavailability due to the identity of chemical properties with the ordinary water molecule [1,2,4–7]. As a consequence, HTO easily enters any biosystem, especially plants. The atmospheric HTO is absorbed by plants through their leaves, where it is directly converted into organically-bound tritium (this definition will be used further on in the article as OBT) during photosynthesis [8]. Over the past decades, the interest in OBT has significantly increased [1,2,9–12] because it has more resistance in biological systems compared to HTO [13,14]. The OBT, which forms in agricultural plants, transports and stores itself in edible parts [15–18]. Thus, the edible parts of crops represent a sink for OBT in agricultural ecosystems.

According IAEA data, for routine releases, tritium transfer to crops have no consequences on any health effects but for accidental release the contribution of OBT for the ingestion dose can be as high as 80% of the tritium dose [19]. It should be noted, a biological half-life of HTO in adults has approximately 10 days, organically-bound tritium will remain in the organism for up to around 40 days [20], and some amount of organically-bound form of the isotope can remain in the fat and collagen tissues even for around 450 days [4]. The studies with administration of D-labelled glucose, alanine, palmitic acid, or soybean into organism of many volunteers suggested that OBT in ingested compounds and food, gives higher doses than the current dose coefficient [21]. Thus, the tritium concentration, especially in OBT crops at harvest, requires attention due to the possible contribution to the public dose in the NFC adjacent territories in case of accidental releases. In this context, the prediction of tritium uptake and its incorporation into edible plants is an important issue. In the opinion of Melintescu & Galeriu [10], it is difficult to clarify which from existing models will be better predict an accidental situation with tritium emission, based only on the experimental data sets for a single crop grown in specific environmental conditions. The consequences of an accidental release of radioactive material will vary significantly depending on the conditions that occurred at the time of the accident, in particular, on prevailing meteorological conditions, time of year, location and habits of the population. In addition, the sensitivity of plant HTO and OBT concentrations to changes in meteorological conditions is currently insufficiently studied [22]. The data available in the literature on the study of air transport of tritium to agricultural crops and its incorporation into organic matter are primarily obtained either under laboratory conditions [23–26] or in in mid-latitudes [27–30]. At the same time, experimental data on the absorption of tritium by crops in the arid climate are practically absent. Obtaining quantitative parameters for tritium uptake by crops in arid climates is essential for Central Asian countries with predominantly agrarian economies. Such data are especially important for Kazakhstan in connection with the construction of a nuclear power station in the near future [31]. In this study, we focused on quantifying the evaluation of tritium uptake in crops and its incorporation into organic matter in the area heavily contaminated by atmospheric tritium oxide (HTO) at the Semipalatinsk Test Site in Kazakhstan.

## Materials and methods

### Plants cultivation

Typical crops cultivated all over the Republic of Kazakhstan were selected as experimental plants: lettuce (*Lactuca sativa* L.), tomato (*Solanum lycopersicum* L.), pepper (*Capsicum Annuum* L.) bean and (*Phaseolus vulgaris* L.). Crops were pre-raised in plastic pots (V = 35 L), with light chestnut loamy soil, in a semi-outdoor condition, until the ripening stage. At this stage, the most active accumulation of tritium occurs in the reproductive part, which is usually edible [26,32]. Therefore, tritium contamination of plants during this growth period is the greatest risk to the public in case of an accidental release. Contamination of plants at earlier stages is less dangerous due to the dilution of up taken tritium by a pure newly formed substance until harvest.

During growth crops were watered with distilled water maintaining an optimal humidity of 60% of full soil moisture capacity. Appropriate amounts of the composite fertilizer were supplied as required during the plant growth. Also, disease and insect controls were carried out as required.

### Experiments with atmospheric HTO crop exposure

**Fig 1:**
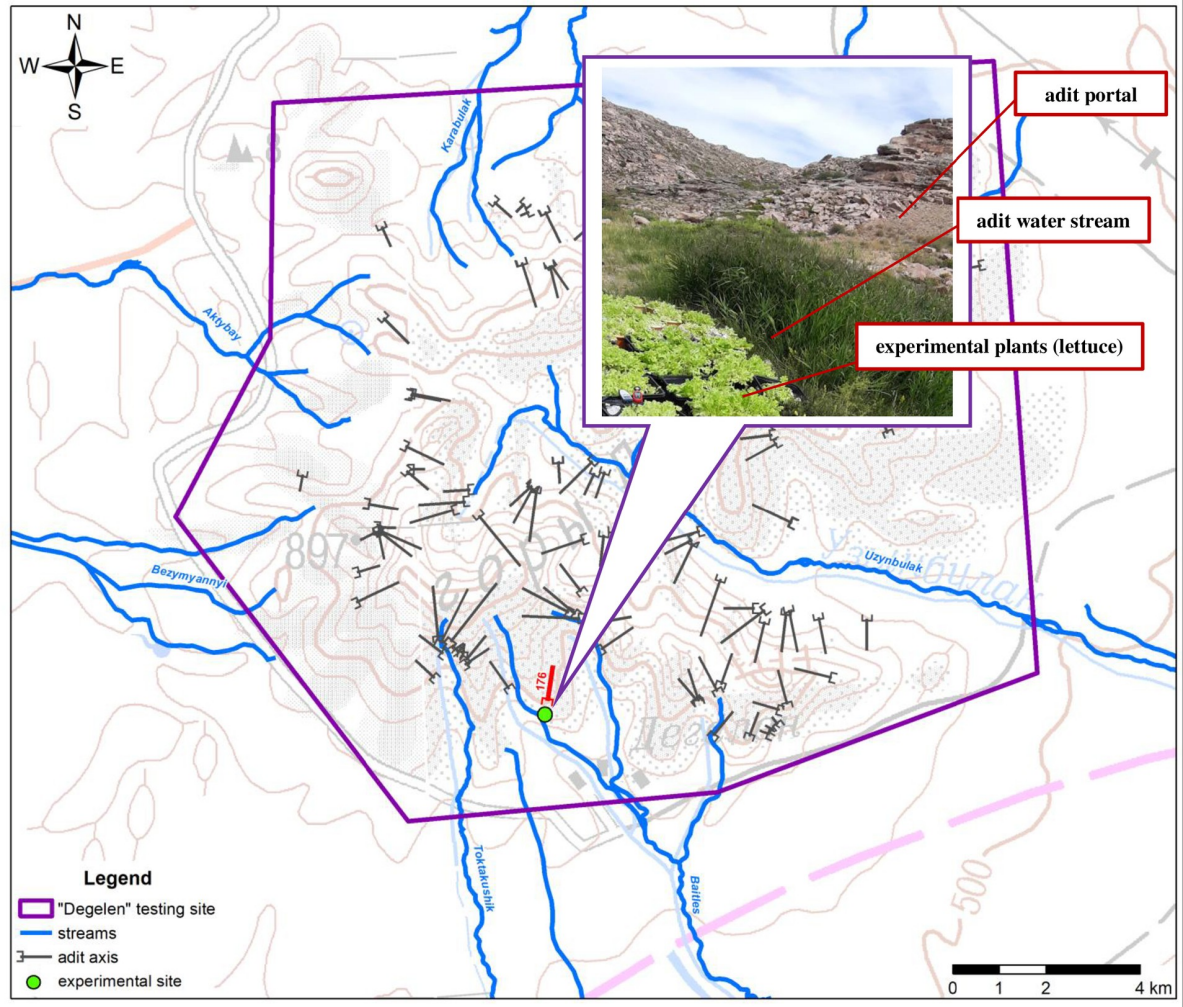
The location of the crop exposure place at the former technical test site "Degelen" (on the photo is lettuce exposure).

For a better reproduction of the actual impact of environmental conditions, experiments were carried out in the territory of the former Semipalatinsk Test Site (STS), namely, at the "Degelen" technical site. At the "Degelen" site, underground nuclear tests were conducted in horizontal adits [33–35]. In this regard, most radioactive substances are concentrated in the cavities of adits. In this regard, most radioactive substances are concentrated in the cavities of the adits. Nowadays, as a result of precipitation, the cavities are filled with water, which then flows out as radioactive-contaminated water streams to the surface [34]. Tritium takes a special place in the radioactive contamination of the natural environment of the "Degelen" site [33–35]. There are a lot of adits at the "Degelen" site, which from outflow radioactive contaminated water streams. In all experiments, the pots with plants were placed near the adit number 176, 1 meter away from the radioactive water stream (Fig 1).

This radioactive water stream was characterized by a high level of HTO contamination, both in the water and in the surface air. The maxima of HTO concentration recorded in the water [34] in the experimental area and in the air [33] were 341 kBq L^-1^ and 1400 Bq m^-3^, consequently. The level of tritium contamination in the water stream and the surface air near the tunnel varies depending on the volume of rainfall over a given area [34].

The branch "Institute of Radiation Safety and Ecology" of the National Nuclear Center of the Republic of Kazakhstan obtained a permission from the State Institution "Committee for Atomic and Energy Supervision and Control" of the Ministry of Energy of the Republic of Kazakhstan (License No. 19014222 dated 07/03/2019) to conduct research at the former STS. Personal protective equipment was used to prevent personnel contamination during the work.

The exposure experiments were conducted during the middle of the summer in 2019, 2020, and 2021. The exposures of plants started between 9:30 a.m. and 10:00 a.m. and ended between 16.00 p.m. and 18.00 p.m. Thus, during the exposure in all experiments, the diurnal rhythms of plants matched. This condition is important, since most physiological processes in plants, in particular, resistance of stomata and photosynthetic activity, are linked to daily biorhythms [36]. Plant exposure lasted from 6 to 8 hours. During the exposure, the soil in the pots was covered with polyethylene film to prevent HTO from being diffused into the soil and, accordingly, from being absorbed by roots. After exposure, the plants were left to grow naturally in a semi-outdoor condition for 14 days while being irrigated with distilled water.

During the experiments, temperature, relative humidity, and atmospheric pressure were recorded at 1 h interval using a thermal hygrometer (IVA-6, Russia). The light intensity was measured using a spectrometer (TEK, Russia). The values of each parameter were calculated as an average of three measurements taken every hour.

### Sampling method

#### Plant sampling

Plant sampling was carried out during the exposure at 2 h intervals (2, 4, 6, and 8 hours after initiation of exposure), and then at 1, 24 and 336 h (14 day) after the end of exposure. Plant shoots in quantity of 5–7 were randomly collected and divided into parts: leaves, stems and fruits. In the case of lettuce, the above-ground part was collected. All plant samples were collected in 3–4 replicates. Therefore, small plant samples can be considered representative. To prevent the loss of tritium in the field, plant samples were immediately packed in zip bags and stored in a portable freezer at -20°C. The tritium concentration was measured in free water and organic matter of plants.

#### Air sampling

Air samples were collected at 2 h intervals using a tritium collector "OS 1700" (AMETEK, USA). The average sampling duration for the tritium collector "OS 1700" is 2 hours. The tritium collector was placed next to the pots with plants. The size of the site where the plants and collector were located was 3×3 meters on average, which eliminated significant variation in the concentration of НTO values in surface air due to wind, turbulence, and solar energy flow. The volume activity of tritium in each air sample was calculated as the average of the measurement results from 3 counting samples made from the initial sample.

### Analytical methods

#### The measurement of tissue-free-water tritium in plant samples

**Fig 2:**
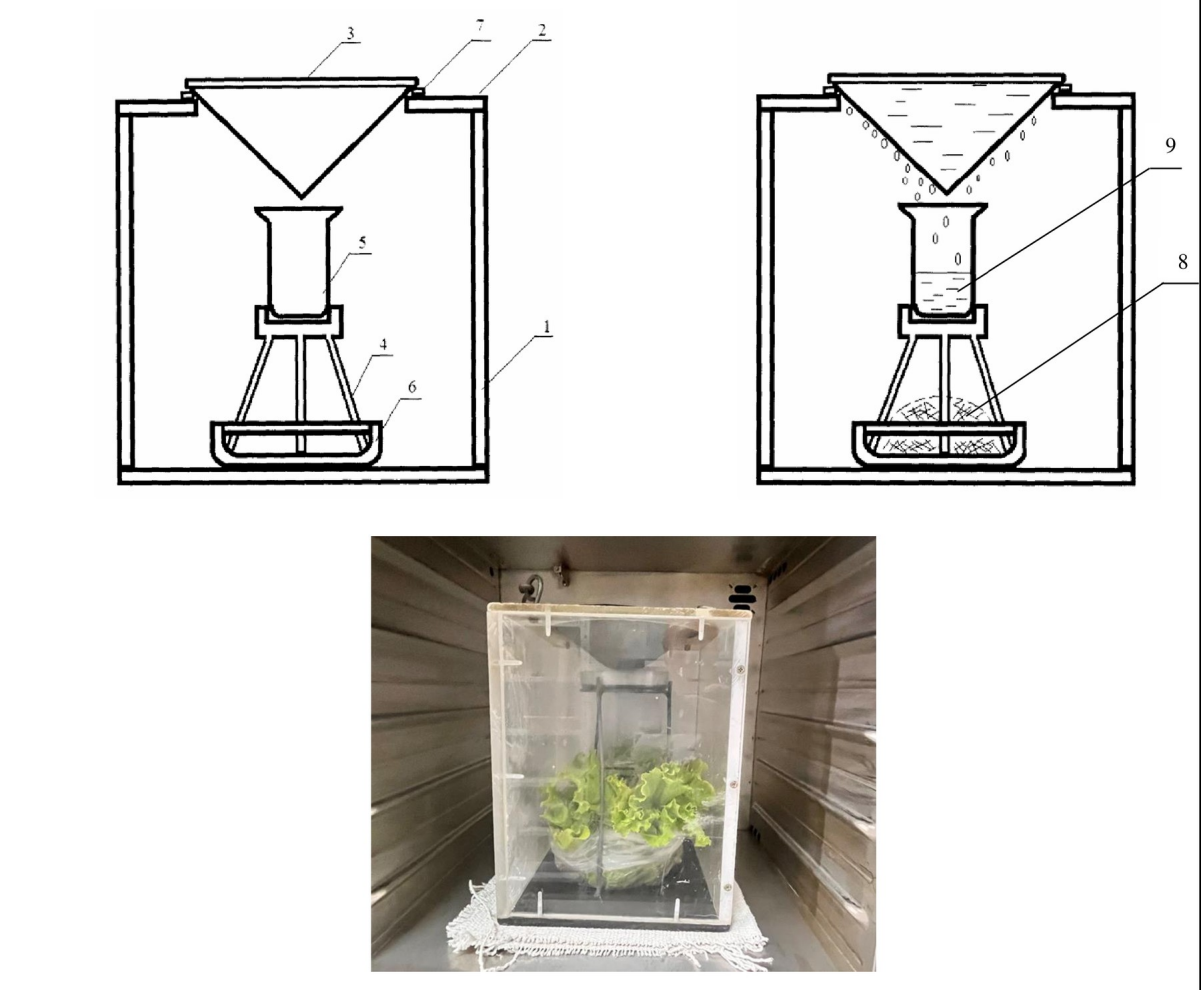
Scheme [37] and photo of a device for extracting tritium from plants’ tissue free water (1 –organic glass container; 2 –lid; 3 –cone-shaped refrigerator; 4 –receiving tank holder; 5 –receiving tank; 6 –sample container; 7 –sealer; 8 –plant sample; 9 –condensate containing plants’ free-water tritium).

The tissue-free-water tritium (TFWT, or HTO) extracted from plant samples using a special device [37] (Fig 2).

Samples of plant were put into transparent box, which had a cover in the form of a cooling vessel. In this device cold water was used as a cooling agent. By natural evaporation of the plant sample, free water condensate was obtained from the plant tissues. The obtained condensate corresponded to tritium as a tritiated water named "tissue-free-water tritium" (this definition will be used further on in the article as TFWT). The extracted TFWT was transferred to a counting polyethylene vial. The volume of condensate varied from 10 to 15 ml. The TFWT activity concentration was measured by liquid scintillation spectrometry.

#### The measurement of organically-bound tritium in plant samples

For the determination of OBT in plant samples, the combustion method was used. After tissue-free water was extracted, the plant samples were dried in a laboratory drying oven (BINDER ED 53, Germany) at a temperature of 70–80°С. The dried samples of plant parts were powdered using a laboratory mill (GRINDOMIX GM 200, Germany). About 1.0 to 2.0 g of the dry powder was weighed and combusted with a "Sample Oxidizer" (PerkinElmer Model No. 307, USA). Water obtained after combustion was collected in a tritium counting vial. The ОВТ activity concentration was measured by liquid scintillation spectrometry.

#### Liquid scintillation counting

Tritium activity concentration was measured by liquid scintillation spectrometry using a "QUANTULUS 1220" spectrometer (Perkin Elmer, USA) [38]. Prior to the measurement, samples were filtered to remove mechanical impurities, then a 3 ml aliquot was collected into a 20 ml plastic vial and an Ultima Gold LLT scintillation cocktail for natural samples (the registration efficiency for tritium in the 0–18 keV range of about 60%) was added at a ratio of 1:4 ("sample-scintillator" ratio). The measurement time was at least 120 minutes, the beta spectra were processed and the activity concentration of tritium was calculated using the program "Quanta Smart". The minimum detectable activity (MDA) for HTO is approximately 4–5 Bq l^-1^. The minimum detectable activity (MDA) for OBT is approximately 8–10 Bq kg^-1^.

The volumetric activity of tritium in each air sample was determined as the arithmetic mean from measurements of 3 counting samples prepared from the initial sample.

#### Calibration of the "QUANTULUS 1220" beta spectrometer

The calibration of the "QUANTULUS 1220" spectrometer was carried out using reference tritium sources with varying degrees of quenching to determine the efficiency of registration. The exposure time of the reference samples was 10 minutes. The calculation of the registration efficiency was performed using the following formula:

$$Ɛ=\frac{N}{A_{Cal}}\times e^{-\frac{\left(t-T\right)}{12.3}}$$ (1)

N–the count rate in the registration window, minus random coincidences, CPM (count per minute);

*A*_*Cal*_*−*the activity of the source at the time of certification;

*t–*the year at the time of calibration;

*Т–*the year of certification of the source.

Based on the data obtained, SQP quenching parameters were determined for each standard, and a graphical dependence of registration efficiency on quenching parameter was constructed. The frequency of updating the quenching curve is at least once a year.

#### Mapping

The maps presented in this article were created using ArcGIS software based on digitized maps of Kazakhstan purchased from the National Cartographic and Geodetic Fund of the Ministry of Digital Development, Innovation and Aerospace Industry of Kazakhstan.

#### Quality control

The stability of analytical results was overseen in the form of a random statistical control (by an alternative sign) within the laboratory accuracy of analytical results.

The reproducibility of analytical results for each series of samples was overseen by 2 parallel tests of one working sample including sample preparation and analysis. After the analysis, the deviation from the mean was calculated by the formula:

$$\Delta A=\left|\frac{A_{1}-A_{2}}{A}\right|$$ (2)

A_1_ и A2—activities of the first and second samples;

А–arithmetic mean.

The maximum permissible deviation from the mean is assumed to be 0.5. If this condition is fulfilled, the reproducibility of parallel determinations is considered satisfactory.

Each series of working samples was followed by a blank sample. The results of the analysis of blank samples were considered acceptable if the activity concentration of the radionuclide was below the detection limit.

## Results and discussion

### Exposure conditions

Table 1 provides data on the meteorological conditions during each exposure. The light intensity is presented in PPFD (density of the photosynthetic photon flux).

**Table 1:** Meteorological conditions during exposures.

Exposure cod	n	Temperature, °C		Relative humidity, %		PPDF, μmol m^-2^ s^-1^	
		mean±SD	min–max	mean±SD	min–max	mean±SD	min–max
**L**	24	30±7	21–35	85±3	81–88	1489±172	1275–1695
**L+T+B**	24	24±4	20–28	52±9	41–63	875±209	345–1590
**P**	24	28±5	23–37	63±9	55–80	555±201	219–1506

^a^ error bar shows S.D. of six values.

^b^ L–lettuce exposure, T–tomato exposure, B–bean exposure, P–pepper exposure.

As shown by Table 1, the mean values of air temperature ranged from 24 to 30°C. The mean values of relative air humidity varied from 52 to 85%. The density of the photosynthetic photon flux significantly changed during exposures from hundreds to thousands μmol s^-1^ m^-2^. The highest value of PPFD were observed for lettuce exposure in 2020 year.

**Fig 3:**
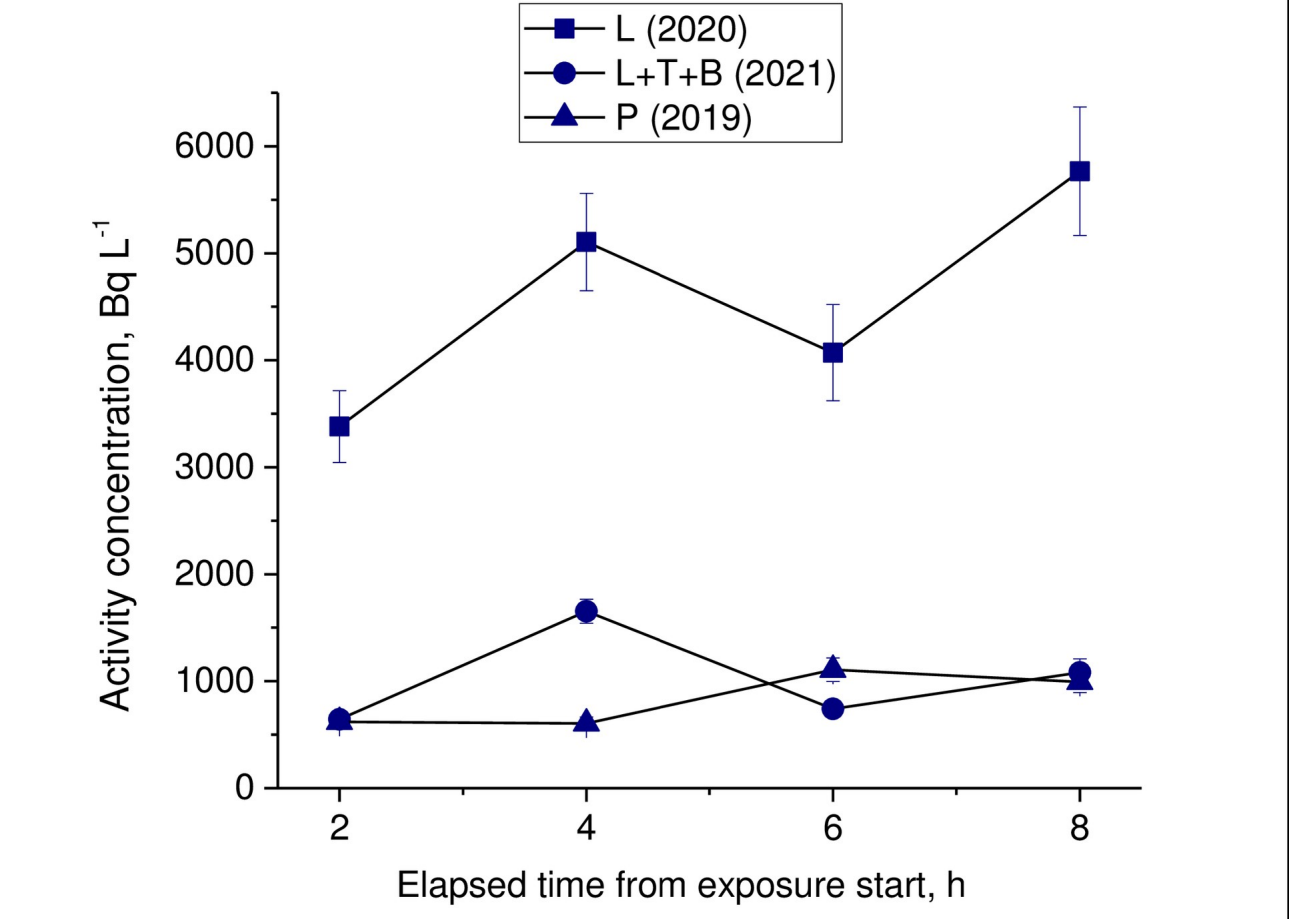
Variation in HTO concentration in air moisture during each exposure (L–symbolizes lettuce exposure, T–symbolizes tomato exposure, B–symbolizes bean exposure, P–symbolizes pepper exposure; error bar shows S.D. of three replicates).

At the former test site during research HTO concentration of in the surface air for the varied over a wide range. The results of measuring the concentration of HTO vapors at each exposure are shown in Fig 3.

In 2019, when pepper plants were exposed HTO concentration was minimal and averaged (762±86) Bq L^-1^). In 2020, when experiment with lettuce was conducted the concentration of HTO in the atmospheric air was maximum and averaged (4580±560) Bq L^-1^. In 2021, during exposure of tomatoes, beans and lettuce the average concentration of HTO vapors was (903±110) Bq L^-1^.

### TFWT in plants

**Fig 4:**
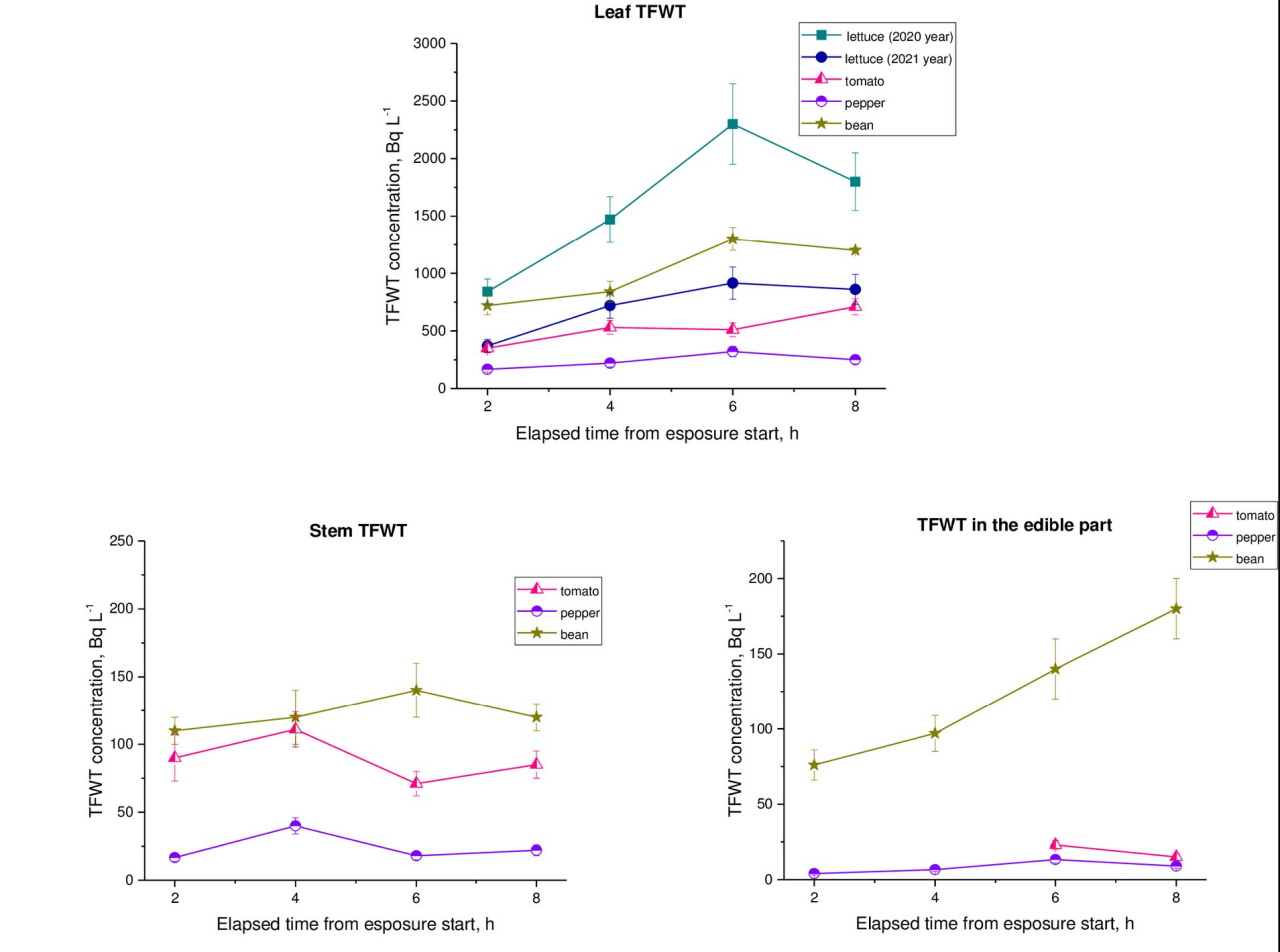
Time courses of TFWT activity concentration in plants during exposures.

Fig 4 shows the average values of TFWT activity concentrations in crops during experiments.

As shown in Fig 4, 2 hours after the start of each exposure, the concentrations of TFWT in plant leaves and HTO in ambient air were in the same order. This fact is undoubtedly due to the direct entry of HTO through the leaves as a result of vapor exchange [1,2,5]. The TFWT concentration in the stems and edible parts of the plant was 1 to 2 orders of magnitude lower than in the leaves and in ambient air. This is because these plant parts have a lower rate of water exchange with the atmosphere compared to leaves. Mainly, HTO is transported to the stems and the edible parts through the phloem [29]. In most cases the distribution of concentration TFWT was as follows: leaf–stem–fruit (the descending series). The obtained data is in agreement with the results of rice exposed to atmospheric HTO vapor exposed to atmospheric HTO vapor in semi-outdoor conditions [26], and wheat exposed in a box [24]. In the field experiments conducted by Kline and Stewart [27], the TFWT activity concentration in crop leaves was also an order of magnitude higher than in other plant parts during exposure to atmospheric HTO. The results of the experiments by Koranda *et al*. [39] with sunflower and red oat, Guenot and Below [28] with potato and grape, Choi *et al*. [26] with rice, and Polivkina *et al*. [32] with pepper and eggplant observed a similar pattern.

For comparison, the concentration ratio TFWT/HTO_atm_ was calculated during exposure which reflects the relative content of tritium in the free water of plants to atmospheric HTO (Table 2).

**Table 2:** TFWT/HTO_atm_ ratios in plant parts during the exposure.

h	TFWT/HTO_atm_ ratio (mean±SD^a^)				
	lettuce (2020 year)	lettuce (2021 year)	tomato (2021 year)	bean (2021 year)	pepper (2019 year)
leaf					
2	*	*	0.5±0.1	1.0±0.1	0.5±0.10
4	*	*	0.5±0.1	1.1±0.1	0.4±0.10
6	*	*	0.5±0.1	0.8±0.1	0.3±0.04
8	*	*	0.6±0.1	1.2±0.02	0.3±0.04
stem					
2	*	*	0.13±0.02	0.1±0.01	0.049±0.010
4	*	*	0.14±0.01	0.2±0.02	0.066±0.010
6	*	*	0.11±0.01	0.1±0.02	0.016±0.003
8	*	*	0.07±0.01	0.1±0.02	0.022±0.004
edible part					
2	0.3±0.03	0.6±0.1	–	0.1±0.01	0.012±0.002
4	0.3±0.04	0.7±0.1	–	0.1±0.01	0.011±0.002
6	0.6±0.1	0.9±0.1	0.02±0.003	0.1±0.02	0.012±0.002
8	0.3±0.04	0.7±0.1	0.01±0.002	0.1±0.02	0.009±0.002

^a^ "SD"–the error shows the standard deviation of the 3–4 replicates.

^b^ *–for lettuce, the above-ground part is the edible part.

^c^ "–" data no obtained.

As can be seen from Table 2, the TFWT/HTO_atm_ ratio for crop leaves in the experiments in 2019, 2020, and 2021, varied widely from 0.3 to 1.2. The ratio of leaves TFWT to atmospheric HTO averaged within all experiments over the 3 years is (0.73±0.2). The obtained values agree well with literature data. So, in experiments with wheat, Diabate and Strack [24] reported that the concentration of TFWT in the leaves relative to the concentration of HTO in air moisture at the end of exposure was 0.75–0.86. The TFWT/HTO_atm_ ratio in Chinese cabbage and rice leaf after short-term exposure was 0.24 and 0.35, respectively [40]. The wide range of variability in the ratio in this study is clearly due to differences in meteorological conditions between different years (Table 1). It is the meteorological conditions that determine the conductance of the stomata and, accordingly, the intensity of aerial absorption of tritium by plants. On the other hand, HTO absorption certainly depends on the morphophysiological characteristics of the plant, such as leaf plate structure and average number of stomata. The obtained TFWT/HTO_atm_ ratios for beans, tomatoes, and lettuces, simultaneously exposed to atmospheric HTO under the same meteorological conditions, confirm this (Table 2). So, the results of the experiments by Belot *et al*. [41] with wine grape showed that the diffusion of tritium through the stomata is actually a limiting factor in the movement of tritium from the air into the leaves of plants, even when the stomata are wide open. In experiments using deuterium oxide as a substitute for tritium oxide [42], different uptake rate of isotope was also observed in radish, cherry tomato, komatsuna, and orange due to differences in stomatal resistance.

The values of TFWT/HTO_atm_ ratio averaged over all experiments for 3 years were (0.09±0.003) for crop stems and (0.04±0.002) edible parts. This may be due to the fact that the surface-to-mass ratio is much lower in stems and the edible part than in leaves, leading to a lower content of TFWT. Similar pattern has been reported by other authors during short-term exposure [24–26].

**Fig 5:**
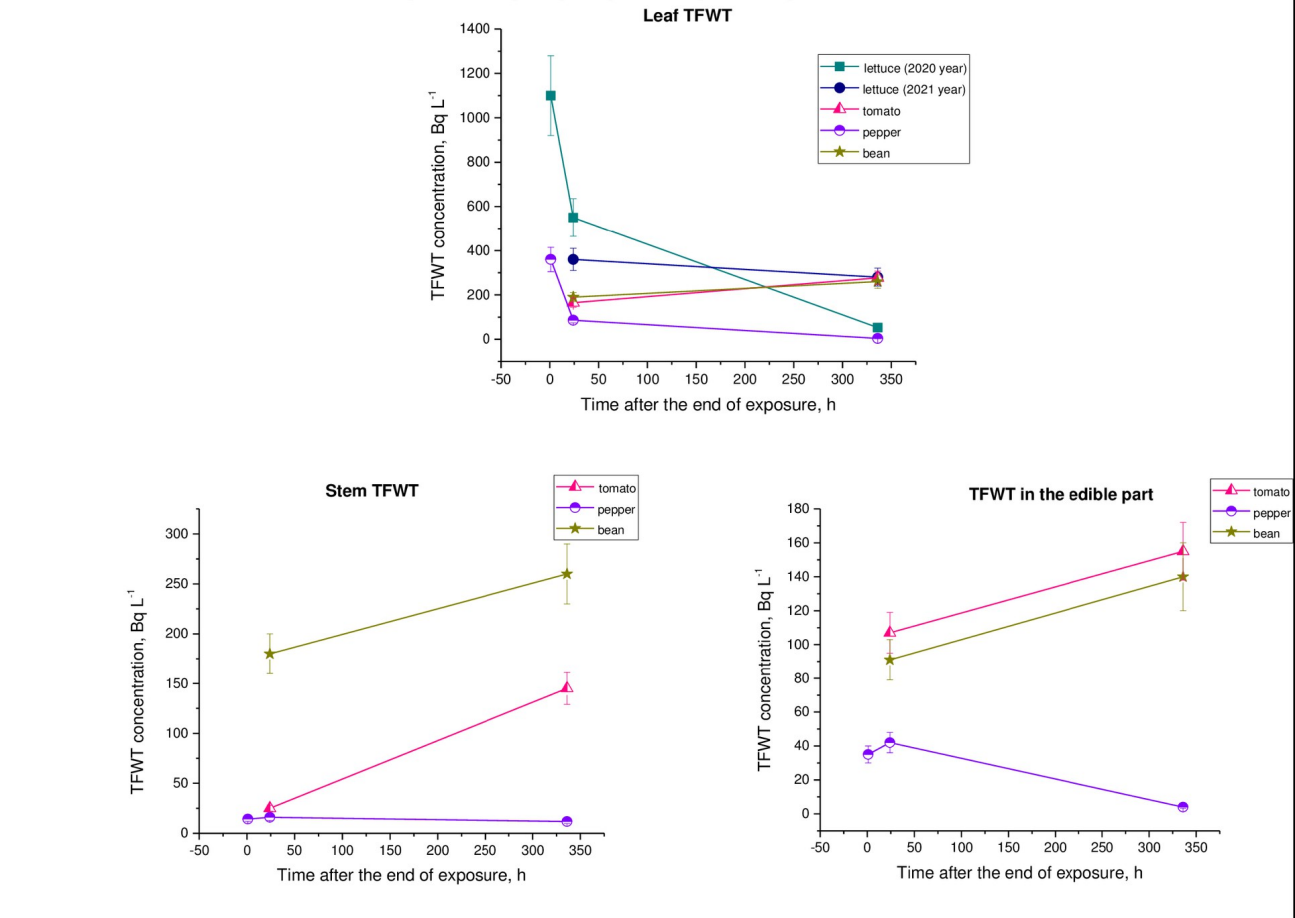
Time courses of TFWT activity concentration in plants after exposure.

Fig 5 shown the dynamic of TFWT loss in plant parts after exposures.

After the end of each exposure, the concentration of TFWT in leaves decreased rapidly during the first few hours, and then at a much slower rate over the next 336 hours (14 days). The sharp decrease in the concentration of TFWT leaves in the first hour and day was not particularly unexpected, due to the rapid exchange of this compound between leaves and the atmosphere. In experiments with cabbage plants and lettuce [29], Chinese cabbage and rice [40] a rapid decrease in the TFWT concentration of leaves was also observed after atmospheric exposure to HTO. The TFWT concentration at harvest was 3 to 90 times lower than it was at the end of the exposure period. In the rice experiment, this difference was between 600 and 95000 times [26]. This may be due to the fact that the atmospheric HTO concentration during the rice exposure period was significantly higher and the time of exposure was significantly shorter.

The rates of TFWT loss in lettuce leaves in 2020 and 2021 (Fig 3) differed by an order of magnitude. This can certainly be explained by the difference in the concentration of atmospheric HTO during exposure and meteorological conditions during actual decline. It is well known that transpiration is one of the main causes of plant HTO loss [27,29]. This process depends on factors such as temperature, relative humidity, and solar radiation intensity, and other factors that affect stomatal resistance. The differences in the rate of TFWT loss in leaves of tomato, bean and lettuce during exposure in the same conditions can be explained by the physiological characteristics of these plants. These include the structure of leaf blades, the number of stomata and even the strength of their root suck. The results of the rice experiment obtained by Choi *et al*. [26] also indicate that the loss of TFWT may be determined by physiological and meteorological conditions.

In the stems and edible parts of tomatoes and beans, on the contrary, there was an even slight increase in TFWT concentration after exposure, which is probably due to TFWT outflow from the leaves. The concentration of TFWT in the stem of pepper practically did not change over 14 days. However, in the edible part, it increased in the initial days, followed by a slow decrease. It is obvious that such dynamics of TFWT is also explained by the processes of redistribution of tritium as a result of xylem transport of water in plants.

A small fraction of the TFWT was tenaciously retained for a long time, probably due to the slow dilution of colloidal water and regeneration of the TFWT from OBT. The concentration of TFWT in the leaves has become almost equal to that in the stems and edible part 14 days after the end of exposure. In experiments with rice [26], cabbage [25], wheat [24], tomatoes, corn, and potatoes [12], after the end of each exposure, the concentration of TFWT in the leaves also decreased rapidly during the first few hours, and at a much slower rate during the subsequent, longer period.

To compare of TFWT loss rate from the plant tissues the reduction factor has been applied (Table 3).

**Table 3:** Reduction factor in different plant parts after exposure (14 days).

Code of exposure	Exposure time	Reduction factor^a^ (mean±SD^b^)		
		leaf	stem	edible part
lettuce	2020 year	34±18.2	**—** ^**c**^	**—**
lettuce	2021 year	3.1±1.7	**—**	**—**
tomato	2021 year	2.6±1.7	1.0±0.6	0.1±0.08
bean	2021 year	4.6±2.8	0.9±0.3	0.7±0.30
pepper	2019 year	2.9±1.7	0.5±0.2	0.8±0.40

^a^ TFWT concentration at the end of exposure.

TFWT concentration at harvest after exposure.

^b^ "D"–the error shows the standard deviation of the 3–4 replicates.

^**c**^ "**—**"–data no obtained.

As may be seen in Table 3, the reduction factor varied by factors of 3–26 depending on plant parts. The reduction factor was greatest in the leaf in every exposure due to vapour exchange and transpiration. The stem and edible part showed the lowest reduction factor for each exposure. This is because of the mechanism of decontamination of these plant parts occurs through the phloem. These mechanisms of TFWT loss have previously been considered by Brudenell *et al*. [29]. A similar pattern was observed by Indeka [9] in experiments with tomato, potato, sunflower and corn, Diabate and Strak [24], Strack *et al*. [15] in experiments with wheat, and Choi *et al*. [25] in experiments with rice.

However, the reduction factors in our study were significantly lower than in rice [26], wheat [24], sunflower and maize [12]. Obviously, this is due to the higher concentrations of HTO during the experiments of the aforementioned authors.

### Tritium incorporation by plants

Table 4 provides mean values of OBT activity concentration in plants during and after exposures.

**Table 4:** OBT activity concentration in plants during and after exposure.

Cod exposure/ year	part of plant	OBT concentration (Bq L^-1^) mean±SD^a^						
		exposure time (h)				post-exposure time (h)		
		2	4	6	8	1	24	336
lettuce (2020)	edible part	22±5	93±20	68±15	78±15	75±12	57±12	28±8
lettuce (2021)	edible part	42±8	42±8	74±15	77±13	—	89±16	78±13
tomato (2021)	leaf	167±50	200±58	142±40	170±43	192±45	192±45	68±25
	stem	75±25	158±43	78±27	110±33	78±25	60±25	60±22
	edible part	—	—	65±30	92±40	—	153±47	83±40
pepper (2019)	leaf	12±5	37±10	30±7	33±8	43±8	67±15	10±4
	stem	8±1	13±5	7±4	18±7	15±5	12±5	15±5
	edible part	8±1	23±7	20±7	15±7	6±3	10±5	8±4
bean (2021)	leaf	28±8	30±10	63±22	35±15	—	75±17	52±13
	stem	—	13±3	15±5	15±5	—	—	—
	edible part	13±3	30±10	28±8	75±17	—	—	53±13

^a^ "SD"–the error shows the standard deviation of the 3–4 replicates.

According to Table 4, concentrations of OBT were 1 to 2 orders of magnitude lower throughout all experiments than TFWT. This is due to the organic bound form of the radionuclide being the result of biochemical processes, which requires more time than the diffusion of HTO through stomata. In addition, OBT formation depends on the physiological state of the plant and photosynthesis rate [1,2,8,11].

Due to the variability in HTO concentrations in the air (Fig 3) and meteorological conditions between experiments (Table 1), the OBT concentrations (Bq L^-1^) were normalized in plant parts to relative values by relating the concentration of HTO in the combustion water to the leaf TFWT concentration (Bq L^-1^). This is based on the knowledge that photosynthesis in leaves is the main source of plant OBT [8,10,11].

**Fig 6:**
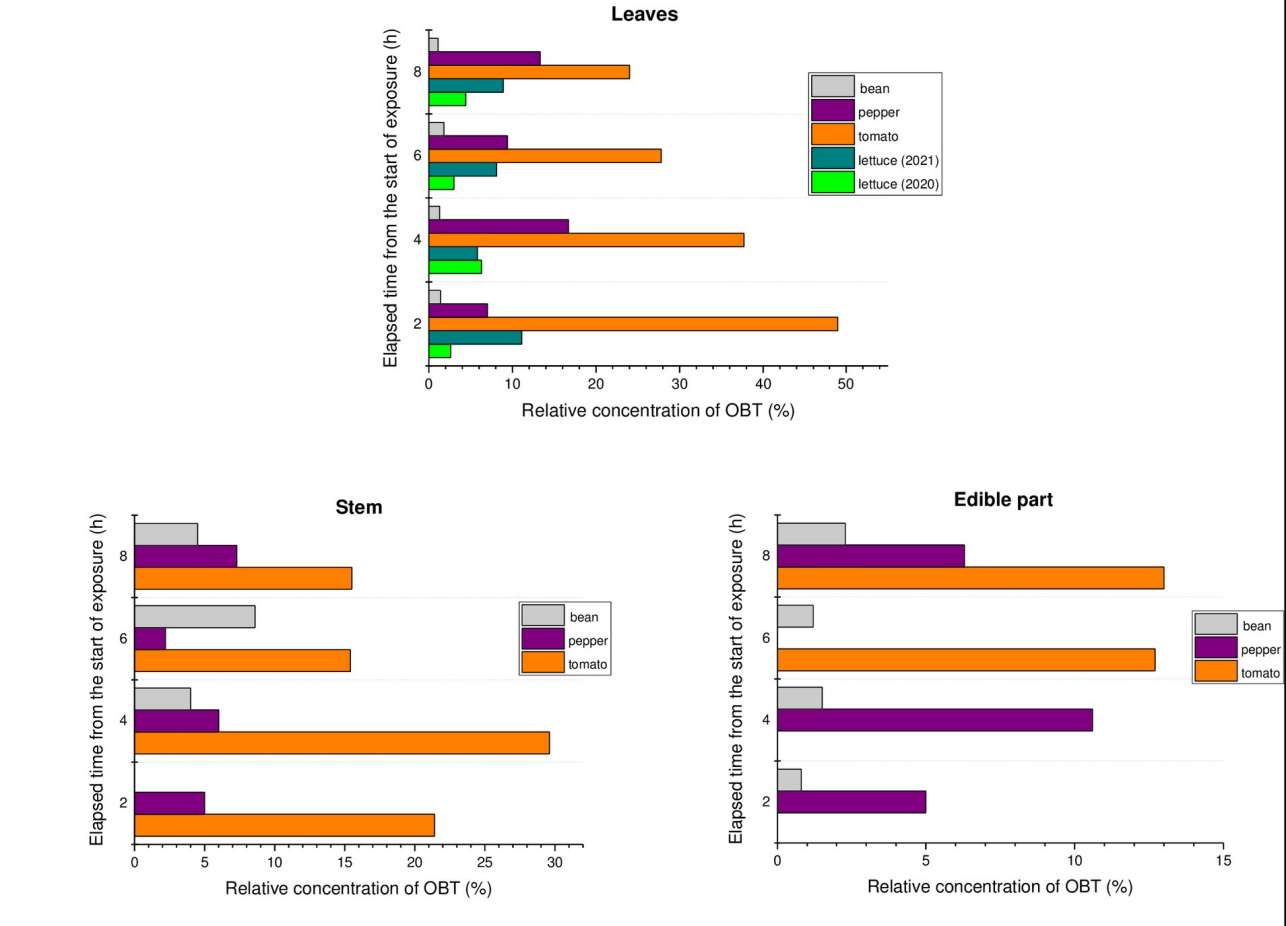
Time courses of relative OBT concentration in plant parts during exposer to atmospheric HTO.

In Fig 6, the relative concentrations of OBT in plant parts are presented during exposure to atmospheric HTO.

According to Fig 6, among crops, the highest concentration of OBT has been observed in the edible part of tomato, while the lowest concentration has been found in beans. This is obviously due to species features such as photosynthesis rate, the speed of translocation of assimilates, the weight, and the biochemical composition of the fruits. So, beans are characterized by a high protein content, while tomatoes contain more carbohydrates. As is well known, more than 90% of the primary organically-bound tritium is formed through photosynthesis and is represented by tritiated simple sugars [1,2,8,10,11,16], which are directly translocated from the leaves to the edible part. Tritiated proteins are formed during secondary metabolic reactions and then transported to beans.

The organically-bound tritium was distributed in non-leafy crops as follows: leaf–stem–fruit (in decreasing order). The maximum concentrations of OBT in the leaves are due to the photosynthetic origin of this form of tritium. The results obtained are in good agreement with the literature data for non-leafy crops [15,24,25,32,40,42].

There was no definite pattern in the dynamics of the relative OBT concentration in all parts of the plants, as both its increase and decrease were observed during the exposure period. The absence of a clear pattern is obviously due to the continuous phloem transport of tritium assimilates formed during exposure. It is possible that dynamic variation in HTO is an additional factor contributing to uncertainty regarding OBT concentrations in plants.

As seen in Table 4, for the first hour and day after exposure, OBT leaf concentrations increased in most cases by 1–2 times compared to the end of exposure or decreased slowly. Diabate and Strak [24] reported that the total amount of OBT in wheat leaves increased until day 1 after exposure. Indeka [9] reported a slow decrease in the concentration of OBT in the leaves of tomatoes, potatoes, sunflower, maize, and wheat. The uncertain dynamic of OBT concentration is due, firstly, to the fact that tritium remaining in free water of leaves continues to incorporate into organic matter. Secondly, the transport of OBT through phloem continues.

In the first hours after the exposure period, the OBT concentrations in stems and edible parts decreased slowly. In the long-term post-exposure period, the concentration of OBT in these plants parts was kept approximately at the same level, and it was more or less similar to that in leaves. This fact can be explained by the ongoing synthesis and transport of OBT through the phloem from the leaves.

To compare the results of experiments the OBT concentration (Bq L^-1^) in each part after exposure was normalized by relating it to the leaf TFWT concentration at the end of the exposure (Bq L^-1^). Table 5 presents the normalized values of OBT concentration in plant parts in post-exposure period.

**Table 5:** Normalized values of OBT concentration in plant parts during the post-exposure period.

Time (h)	Normalized values of OBT concentration (%)										
	L_2020_	L_2021_	T	P	B	T	P	B	T	P	B
	leaf					stem			edible part		
1	4.2	—	—	17.3	—	—	6	—	—	2.5	—
24	3.1	10	27	26.7	2.3	11	4.7	—	21.6	4	—
336	1.6	9.1	9.3	4	1.6	8.5	6	—	11.7	3.5	1.6

^a^ L–lettuce, T–tomato, P–pepper, B–bean.

As can be seen from Table 5, after exposure the relative OBT concentrations in the edible parts at harvest were in the range of 2.5–21% after 1 day, and in the range of 1.6–11% after 14 days, depending on the plant. In the rice experiments [26] the relative OBT concentrations in ears at harvest were in the range of 0.02–0.34% depending on the exposure time. The higher values of relative OBT concentrations in plants in this study may be due to differences in HTO levels during exposure and a longer time of exposure.

It is well known that in the case of HTO release in the atmosphere, the main pathway of exposure is consumption of food with a contribution of tritiated organic molecules (OBT) elaborated by photosynthesis [1,2,10–12,16]. The obtained quantitative data on tritium uptake by leafy and non-leafy vegetables can be used to make a conservative estimate of the possible contribution of the isotope to the public internal exposure dose from ingestion of contaminated crop products in the event of a short-term accidental release of HTO on the territory of Kazakhstan.

### Relation between levels of the atmospheric HTO, tissue-free-water tritium, organically-bound tritium and meteorological factors

The results of the correlation analysis between TFWT, OBT, atmospheric HTO and environmental factors such as temperature, relative air humidity and solar radiation are provided in Table 6.

**Table 6:** The correlation between atmospheric HTO, tritium in plant leaves and meteorological factors.

Variable (N = 22)	C_TFWT leaf_	C_OBT leaf_	C_НТО_	T	PPDF	*φ*
^**a**^ **C**_**TFWT leaf**_	1.00	-0.14	0.76*^f^	0.43*	0.56*	-0,43*
**C**_**OBT leaf**_		1.00	-0.09	-0.21	-0.02	0.01
**C**_**НТО**_			1.00	0.41	0.57*	-0.26
^**b**^ **T**				1.00	0.50*	-0.74*
^**c**^ **PPDF**					1.00	-0.53*
^**d**^ ***φ***						1.00

^a^ C_НТО,_ C_TFWT leaf,_ C_OBT leaf_ are in Bq L^-1^.

^b^ T is in °C.

^c^PPDF in μmol s^-1^ m^-2^.

^d^*φ* is in %.

^f^ *—marked correlations are significant at *p* < 0.05.

According to Table 6, the concentration of tritium in the free water of leaf tissues is closely correlated with the concentration of atmospheric HTO (*r* = 0.76). This is obvious, as HTO penetrates into the leaf via the stomata through diffusion until reaching a steady state. A weak (*r* = 0.43) and moderate (*r* = 0.56) positive correlation was established between the concentration of TFWT, temperature, and intensity of solar radiation, respectively. Between the concentration of TFWT and relative humidity was established a weak negative correlation (*r* = -0.43). These meteorological factors are indirectly related to TFWT. They affect the conductivity of stomata, and accordingly, the flow of HTO into the leaf. However, a reliable correlation was not found between the concentration of leaf OBT and the concentration of atmospheric HTO, the concentration leaf TFWT, and meteorological parameters such as temperature, humidity, and solar radiation. This fact is explained by the biochemical origin of the organically bound form of tritium. As is known, the rate of formation of OBT, first of all, depends on the physiological state of the plant, the stage of growth, as well as species characteristics [10,11].

The results of the correlation analysis between atmospheric HTO and concentrations of TFWT and OBT in different plants parts are provided in Table 7.

**Table 7:** The correlation between atmospheric HTO and tritium in different plants parts.

Variable (N = 22)	^a^C_TFWT leaf_	C_OBT leaf_	C_НТО_	C_TFWT edible part_	C_TFWT stem_	C_OBT stem_	C_OBT edible part_
^**a**^ **C**_**TFWT leaf**_	1.00	0.51	0.71*^b^	0.78*	0.95*	0.51	0.63
**C**_**OBT leaf**_		1.00	-0.06	-0.09	0.46	0.99*	0.98*
**C**_**НТО**_			1.00	0.83*	0.62	-0.09	0.05
**C**_**TFWT edible part**_				1.00	0.81*	-0.10	0.03
**C**_**TFWT stem**_					1.00	0.45	0.57
**C**_**OBT stem**_						1.00	0.98*
**C**_**OBT edible part**_							1.00

^a^ C_НТО,_ C_TFWT leaf,_ C_OBT leaf_ are in Bq L^-1^.

^b^ *—marked correlations are significant at *p* < 0.05.

As can be seen from Table 7, the concentration of tritium in the free water of the edible part of plants is closely correlated with the concentration of atmospheric HTO (*r* = 0.83) and leaf TFWT (*r* = 0.78). The concentration of TFWT in stems correlates strongly with the TFWT of leaves (*r* = 0.95), and closely with that of the edible parts of plants (*r* = 0.81). The concentration of OBT in the leaves is strongly correlated with OBT in the stems (*r* = 0.99), as well as in the edible parts (*r* = 0.98). A significant functional relationship between the concentrations of tritium in different parts of plants is due to the fact that they are connected by a phloem transport. The obtained correlations are consistent with the approach of the modified SA (specific activity) model recommended by the Canadian Standards Association (CSA) for predicting tritium transport in the "air-vegetation" system [43], based on the ratio of tritium concentrations in plants and the air. However, in order to obtain more precisely average values of tritium uptake parameters, it should be necessary to conduct similar studies over a wider range of meteorological conditions that are typical of the territory of Kazakhstan.

## Conclusion

As a result of a series of experiments conducted in an area heavily contaminated with tritium at the former Semipalatinsk test site in Kazakhstan, a quantitative assessment of the HTO uptake by typical crops grown throughout Kazakhstan (lettuce, tomatoes, peppers and beans) after short-term exposure was given. Studies have shown that with short-term aerial uptake of HTO, the concentration of tritium in crops was depended both on the abiotic environmental parameters that determine the conductivity of stomata (temperature, relative humidity, solar radiation) and on the species features of plants. The normalized values of the concentrations of organically-bound tritium showed that the accumulation of the isotope in the organic matter of fruits is more dependent on the species features of plants. The increase in tritium concentration during exposure and the decrease after it occurs faster in leaves compared to fruits and stems due to differences in the mechanisms of isotope loss. During the period after exposure, a small amount of tritium is firmly retained in free water and organic matter of plants for a long time. The obtained quantitative parameters of the aerial uptake of tritium by crops can be used to evaluate the possible contribution to the internal exposure dose of the population living near nuclear power plants through contaminated crop products that have been exposed to short-term HTO air release. The results of the study will contribution to the data on tritium transfer in the "air-vegetation" system, which is used to verify existing models of tritium migration.

## References

[1] Boyer C. et al. Tritium in plants: a review of current knowledge. Environmental and experimental botany. 2009; 67(1):34–51. 10.1016/j.envexpbot.2009.06.008.

[2] Eyrolle F, Ducros L, Le Dizès S, Beaugelin-Seiller K, Charmasson S, Boyer P, et al. An updated review on tritium in the environment. Journal of Environmental Radioactivity. 2018 Jan;181: 128–37. doi: 10.1016/j.jenvrad.2017.11.001 29149670

[3] Tanabe T. (ed.). Tritium: Fuel of fusion reactors.–Tokyo: Springer Japan, 2017.

[4] Okada S, Momoshima N. Overview of Tritium. Health Physics. 1993 Dec;65(6):595–609. 10.1097/00004032-199312000-00001. 8244708

[5] Garland J. A. Transfer of tritiated water vapour to and from land surfaces. Behaviour of tritium in the environment. 1979; pp. 349–358.

[6] Murphy CE. Tritium Transport and Cycling in the Environment. Health Physics. 1993 Dec;65(6):683–97. doi: 10.1097/00004032-199312000-00007 8244714

[7] International Atomic Energy Agency. Tritium in Some Typical Ecosystems. Bernan Press(PA); 1981.

[8] Moses V, Calvin M. Photosynthesis studies with tritiated water. Biochimica et Biophysica Acta. 1959 Jun;33(2):297–312. doi: 10.1016/0006-3002(59)90117-9 13670898

[9] Indeka L. Incorporation of tritiated water from the atmosphere into aqueous and organic components of plants. In: Jaworowski Z, Biological incorporation of Tritium. Final Report to the United States Environmental Protection Agency Concerning Research Contract No. 05-536-3, CLOR-115/D. Warsaw: Central Laboratory for Radiological Protection;1981. pp. 21–27.

[10] Melintescu A, Galeriu D. Uncertainty of current understanding regarding OBT formation in plants. Journal of Environmental Radioactivity. 2017 Feb; 167:134–49. doi: 10.1016/j.jenvrad.2016.11.026 27916298

[11] Kim SB, Baglan N, Davis PA. Current understanding of organically bound tritium (OBT) in the environment. Journal of Environmental Radioactivity. 2013 Dec; 126:83–91. doi: 10.1016/j.jenvrad.2013.07.011 23962797

[12] INTERNATIONAL ATOMIC ENERGY AGENCY. Approaches for Assessing Emergency Situations. Report of Working Group 7 "Tritium Accidents" of the Environmental Modelling for Radiation Safety (EMRAS II). International Atomic Energy Agency Vienna, 2011.

[13] Hunt J, Bailey T, Reese A. The human body retention time of environmental organically bound tritium. Journal of Radiological Protection. 2009 Mar;29(1):23–36. doi: 10.1088/0952-4746/29/1/001 19225188

[14] Etnier EL, Travis CC, Hetrick DM. Metabolism of organically bound tritium in man. Radiation Research. 1984 Dec;100(3):487–502. 10.2307/3576412. 6390489

[15] Ja G, Ameen M. Incorporation of Tritium in Grain Plants. Health Physics. 1979 Jan 1;36(1):35–8. doi: 10.1097/00004032-197901000-00007 422371

[16] Strack S, Diabaté S, Müller J. Raskob W. Organically Bound Tritium Formation and Translocation in Crop Plants, Modelling and Experimental Results. Fusion Technology. 1995; 28:951–956. 10.13182/FST95-A30528.

[17] Diabaté S, Strack S. Organically Bound Tritium. Health Physics. 1993 Dec;65(6):698–712. doi: 10.1097/00004032-199312000-00008 8244715

[18] Inoue Y, Tetsuo Iwakura. Tritium concentration in Japanese rice. Journal of Radiation Research. 1990 Jan 1;31(4):311–23. doi: 10.1269/jrr.31.311 2098551

[19] IAEA, 2008a. Modelling the Environmental Transfer of Tritium and Carbon-14 to Biota and Man. Report of the Tritium and Carbon-14 Working Group of EMRAS Theme 1. Environmental Modelling for Radiation Safety (EMRAS) Programme. International Atomic Energy Agency Vienna. AEA, 2014a.

[20] Eckerman K, Harrison J, Menzel H-G, Clement CH. ICRP Publication 119: Compendium of Dose Coefficients Based on ICRP Publication 60. Annals of the ICRP. 2013 Aug;42(4):1–130. 10.1016/j.icrp.2013.05.003. 23025851

[21] Masuda T, Yoshioka T. Estimation of radiation dose from ingested tritium in humans by administration of deuterium-labelled compounds and food. Scientifc Reports. 2021 Feb 2;11(1):2816. doi: 10.1038/s41598-021-82460-5 33531641 PMC7854751

[22] Korolevych VY, Kim SB. Relation between the tritium in continuous atmospheric release and the tritium contents of fruits and tubers. Journal of Environmental Radioactivity. 2013 Apr; 118:113–20. doi: 10.1016/j.jenvrad.2012.12.004 23337314

[23] McFarlane JC. Tritium accumulation in lettuce fumigated with elemental tritium. Environmental and Experimental Botany. 1978 May;18(2):131–7. 10.1016/0098-8472(78)90010-2.

[24] Diabaté S, Strack S. Organically bound tritium in wheat after short-term exposure to atmospheric tritium under laboratory conditions. Journal of Environmental Radioactivity. 1997;36(2–3):157–175. 10.1016/S0265-931X(97)84985-5.

[25] Choi YH, Lim KM, Lee WY, Park HG, Choi GS, Keum DK, Lee H, Kim SB, Lee CW. Tritium levels in Chinese cabbage and radish plants acutely exposed to HTO vapor at different growth stages. Journal of Environmental Radioactivity. 2005 Apr; 84(1):79–94. doi: 10.1016/j.jenvrad.2005.04.004 15936121

[26] Choi YH, Lim KM, Lee WY, Diabaté S, Strack S. Tissue free water tritium and organically bound tritium in the rice plant acutely exposed to atmospheric HTO vapor under semi-outdoor conditions. Journal of Environmental Radioactivity. 2002 Jan 1;58(1):67–85. doi: 10.1016/s0265-931x(01)00024-8 11763104

[27] Kline JR, Stewart ML. Tritium uptake and loss in grass vegetation which has been exposed to an atmospheric source of tritiated water. Health Physics. 1974 Jun;26(6):567–73. doi: 10.1097/00004032-197406000-00010 4836702

[28] Belot Y, Guenot J, Caput C, Bourdeau F. Incorporation of tritium into organic matter of terrestrial plants exposed to tritiated-water releases of short duration. Health Physics. 1983 Jun;44(6):666–8. 6853193

[29] Brudenell AJP, Collins CD, Shaw G. Dynamics of tritiated water (HTO) uptake and loss by crops after short-term atmospheric release. Journal of Environmental Radioactivity. 1997;36(2–3):197–218. 10.1016/S0265-931X(96)00088-4.

[30] Mihok S, Wilk M, Lapp A, Nadereh St-Amant, Kwamena NOA, Clark ID. Tritium dynamics in soils and plants grown under three irrigation regimes at a tritium processing facility in Canada. Journal of Environmental Radioactivity. 2016 Mar 1; 153:176–87. doi: 10.1016/j.jenvrad.2015.12.025 26773512

[31] Presidential Decree №121 "Validating the Strategy for achieving carbon neutrality of the Republic of Kazakhstan until 2060". Astana 2023, RK (In Russian).

[32] Polivkina EN, Sysoyeva ES, Romanenko EV, et al. Incorporation of tritium by pepper and eggplant cultures with short-term exposure to tritium oxide. Radiatsionnaya Gygiena = Radiation Hygiene. 2022 Jan;15(4):97–105. (In Russian). 10.21514/1998-426X-2022-15-4-97-105.

[33] Lyakhova ON, Lukashenko SN, Larionova NV, Tur YS. Contamination mechanisms of air basin with tritium in venues of underground nuclear explosions at the former Semipalatinsk test site. Journal of Environmental Radioactivity. 2012 Nov; 113:98–107. doi: 10.1016/j.jenvrad.2012.02.010 22672895

[34] Polivkina YeN Larionova NV, Lukashenko SN Lyakhova ON, Abisheva MT Subbotina LF, et al. Assessment of the tritium distribution in the vegetation cover in the areas of underground nuclear explosions at the Semipalatinsk test site. Journal of Environmental Radioactivity. 2021 Oct; 237:106705. doi: 10.1016/j.jenvrad.2021.106705 34329852

[35] Panitskiy AV, Lukashenko SN. Nature of radioactive contamination of components of ecosystems of streamflows from tunnels of the Degelen massif. Journal of Environmental Radioactivity. 2015 Jun; 144:32–40. 10.1016/j.jenvrad.2015.02.021. 25791901

[36] Kosobryukhov AA. Activity of the photosynthetic apparatus at periodic elevation of CO2 concentration. Russian Journal of Plant Physiology. 2009 Jan;56(1):6–13. 10.1134/S1021443709010026.

[37] Innovative patents N° 29721 for the invention, 2015. Device for the Extraction of Water from the Samples. KazInSt Publishing, Astana, p. 122.

[38] Water quality, 2010. Determination of tritium activity concentration. In: Liquid Scintillation Counting Method. International Standard ISO 9698. KazInSt Publishing, Astana, p. 32.

[39] Koranda JJ, Anspaugh LR, Martin R. The Significance of Tritium Releases to the Environment. IEEE Transactions on Nuclear Science. 1972 Feb;19(1):27–39. 10.1109/TNS.1972.4326482.

[40] Kim SB, Lee MH, Choi GS, Choi YH, Lee CW. Investigation into Tritium Behaviour in Chinese cabbage and Rice after a Short-term Exposure of HTO. Journal of Radiation Protection. 1998 Jan; 23(2):75–82.

[41] Belot Y, Gauthier D, Camus H, Caput C. Prediction of the flux of tritiated water from air to plant leaves. Health Physics. 1979 Oct;37(4):575–583. doi: 10.1097/00004032-197910000-00009 536219

[42] Atarashi M, Amano N, Ishimasa M., Ishimasa Y. Deposition of D_2_O from air to plant and soil during an experiment of D_2_O vapor release into a vinyl house. Fusion Engineering and Design. 1998 Sep;42(1–4):133–140. 10.1016/S0920-3796(98)00376-7.

[43] CSA. Guidelines for Calculating Derived Release Limits for Radioactive Material in Airborne and Liquid Effluents for Normal Operation of Nuclear Facilities. Canadian Standard Association. Draft Standard N288.1. 2008.

